# Correlation between DWI-ASPECTS Score, Ischemic Stroke Volume on DWI, Clinical Severity and Short-Term Prognosis: A Single-Center Study

**DOI:** 10.3390/brainsci14060577

**Published:** 2024-06-05

**Authors:** Oana Andreea Dogariu, Veronica Gheorman, Ioan Dogariu, Mihaela Corina Berceanu, Carmen Valeria Albu, Ioana Andreea Gheonea

**Affiliations:** 1 Ph.D. School Department, University of Medicine and Pharmacy of Craiova, Petru Rareș 2 Str., 200349 Craiova, Romania; florescu.oanaandreea@yahoo.com; 2 Department Medical Semiology, University of Medicine and Pharmacy of Craiova, Petru Rareș 2 Str., 200349 Craiova, Romania; 3 Department of Neurology, Emergency County Hospital Targu-Jiu, Progresului 18 Str., 210218 Targu Jiu, Romania; dogariuioan@gmail.com; 4 Department of Cardiology, County Hospital of Craiova, University of Medicine and Pharmacy of Craiova, Tabaci 1 Str., 200642 Craiova, Romania; mihaela.berceanu@umfcv.ro; 5 Department of Neurology, Neuropsychiatry Hospital Craiova, University of Medicine and Pharmacy of Craiova, Calea Bucuresti 99 Str., 200473 Craiova, Romania; carmenvaleriaalbu@yahoo.com; 6 Department of Radiology, Emergency County Hospital Craiova, University of Medicine and Pharmacy of Craiova, Tabaci 1 Str., 200642 Craiova, Romania; ioana.gheonea@umfcv.ro

**Keywords:** acute ischemic stroke, NIHSS, diffusion-weighted imaging, DWI-ASPECTS, stroke volume-DWI, mRS, prognosis

## Abstract

Ischemic stroke is a significant public health concern, with its incidence expected to double over the next 40 years, particularly among individuals over 75 years old. Previous studies, such as the DAWN trial, have highlighted the importance of correlating clinical severity with ischemic stroke volume to optimize patient management. Our study aimed to correlate the clinical severity of ischemic stroke, as assessed by the NIHSS score, with ischemic stroke volume measured using DWI, and short-term prognosis quantified by the mRS score at discharge. Conducted at the largest hospital in Gorj County from January 2023 to December 2023, this study enrolled 43 consecutive patients with acute ischemic stroke. In our patient cohort, we observed a strong positive correlation between NIHSS score and ischemic stroke volume (Spearman correlation coefficient = 0.982, *p* < 0.01), and a strong negative correlation between ASPECTS-DWI score and mRS score (Spearman correlation coefficient = −0.952, *p* < 0.01). Multiple linear regression analysis revealed a significant collective relationship between ASPECTS score, ischemic stroke volume, and NIHSS score (F(1, 41) = 600.28, *p* < 0.001, R^2^ = 0.94, R^2^_adj_ = 0.93). These findings underscore the importance of DWI in assessing ischemic stroke severity and prognosis, warranting further investigation for its integration into clinical practice.

## 1. Introduction

Ischemic stroke ranks as the second leading cause of mortality worldwide, following ischemic heart disease, and remains a significant contributor to disability, particularly in developed nations [1]. Despite advancements in diagnosis and treatment, it continues to pose a substantial burden on healthcare systems globally [2]. Projections indicate a doubling of stroke incidence within the general population over the next four decades, with a notable impact on individuals aged 75 and older [2]. Approximately 80% of strokes result from cerebral ischemia, highlighting the critical importance of accurate diagnosis and timely intervention [3].

This study aims to evaluate the clinical severity of ischemic stroke, as quantified by the National Institute of Health Stroke Scale (NIHSS) score, in relation to ischemic volume and the Alberta Stroke Programme Early CT Score (ASPECTS) calculated from diffusion-weighted imaging (DWI) images. These parameters will be correlated with short-term prognosis, as assessed by the modified Rankin Scale (mRS) score at discharge. Diffusion-weighted imaging (DWI) is the gold standard for detecting the ischemic core, utilizing its sensitivity to the restricted diffusion of water molecules within affected tissues during the acute phase of ischemic stroke [4]. Although the DWI-ASPECT score and ischemic stroke volume measured using DWI sequences are not yet widely employed in clinical practice, their potential utility in informing treatment decisions is increasingly recognized.

Recent studies, such as the DAWN trial [5], have underscored the efficacy of mechanical thrombectomy beyond the traditional therapeutic window, particularly in patients exhibiting clinical-imaging mismatch criteria. These criteria integrate the NIHSS score and ischemic stroke volume measured by DWI or computed tomographic perfusion (CTP) scans, emphasizing the importance of correlating clinical severity with ischemic stroke volume for optimal patient management. In summary, this research seeks to elucidate the relationship between clinical severity, ischemic volume, and short-term prognosis in ischemic stroke patients, leveraging advanced imaging techniques to inform treatment strategies and improve patient outcomes.

### 1.1. Background

Ischemic stroke manifests as the sudden onset of focal neurological deficits resulting from the abrupt interruption of blood flow to the brain. In acute cerebral ischemic injury, the impairment of the energy-dependent Na^+^/K^+^-ATPase pump leads to an osmotic gradient favoring the translocation of water from the interstitial to the intracellular space, resulting in cytotoxic edema. This phenomenon of reduced diffusivity is readily observed in the DWI sequence [6].

The NIHSS (National Institutes of Health Stroke Scale) score serves as a sensitive and reliable tool for quantifying the severity of ischemic stroke. Developed in 1989, it facilitates the prediction of stroke prognosis and aids in establishing realistic treatment goals [7]. The NIHSS encompasses various domains, including consciousness, eye movements, visual fields, facial symmetry, limb strength, sensory function, coordination, language, and spatial relationships, with scores ranging from 0 to 42 [8].

Diffusion-weighted imaging (DWI) relies on MRI to visualize the slow, Brownian motion of water molecules within interstitial and intracellular spaces. This sequence can detect stroke-related abnormalities within minutes of onset, a timeframe where conventional MRI and CT scans may not yet show changes. Reduced water diffusion in infarcted tissue manifests as increased signal intensity in DWI and decreased signal intensity on the apparent diffusion coefficient (ADC) map. This diminished water diffusion is transient and typically normalizes within a week [9].

Studies have shown that DWI lesions during the acute phase represent both the ischemic core and areas of ischemic penumbra, indicating salvageable potential. However, some acute ischemic deficits may present with normal DWI, particularly in posterior territory lesions, necessitating follow-up imaging [10]. The presence of multiple minor diffusion spots on initial MRI correlates with an elevated risk of recurrence [11]. The DWI-FLAIR mismatch has proven valuable in clinical trials, especially in selecting patients for intravenous thrombolysis when the onset of stroke symptoms is unknown [12].

Quantitative assessment of lesion volume with DWI can be performed manually or by using algorithms, which facilitate rapid and accurate volume determination and enable the calculation of the DWI-FLAIR mismatch [13]. The ASPECTS method, developed for evaluating early ischemic changes on non-contrast CT scans, has been adapted for DWI (DWI-ASPECTS). The ASPECTS score is specific to the MCA territory, with lower scores indicating poorer prognosis [14]. Recent studies have demonstrated the potential of automated tools in providing reliable ASPECTS assessments, aiding in timely decision-making [15].

Despite the DWI-ASPECTS score not being commonly utilized to assess ischemic stroke severity or prognosis, recent studies underscore its utility. For instance, it effectively forecasts the short-term prognosis of patients with acute ischemic stroke treated with iv-tPA [16]. Modified DWI-ASPECTS scores have shown superior correlation with volume estimation compared to conventional scores [17], and AI-based calculations have yielded results comparable to manual measurements by experienced neuroradiologists [18].

The modified Rankin scale (mRS) is widely used to evaluate the degree of disability and dependence post-stroke, serving as the primary measure for assessing clinical prognosis in stroke trials [19].

### 1.2. Rationale, Research Question, and Objectives

The rationale for this study stems from the need to better understand the relationships between clinical severity, ischemic stroke volume, and short-term prognosis to enhance patient management and outcomes. Our research question is as follows: how do the NIHSS score, ischemic stroke volume measured using DWI, and ASPECTS score correlate with each other and with the short-term prognosis, as assessed by the mRS score, in patients with acute ischemic stroke?

This study is original and relevant because it aims to provide comprehensive insights into the utility of the DWI-ASPECTS score and ischemic stroke volume in clinical practice. By addressing these goals, our study contributes to the growing body of evidence supporting the integration of advanced imaging techniques in the management of ischemic stroke, ultimately aiming to enhance patient care and outcomes.

The main objective of this study was to highlight the relationship between clinical severity, ischemic volume, and short-term prognosis in stroke patients since there is a growing concern regarding the improvement in these scores. Our research is based on the use of advanced imaging techniques to improve the clinical prognosis of the patient by using proper therapeutic options. The volume of ischemic lesions measured using DWI could be used to help in the optimal evaluation of treatment groups in clinical trials. These correlations represent a valuable tool for clinical practice, as they can be used in clinical trials to exclude patients with a high probability of good or worse outcomes.

Previous studies have shown a correlation between NIHSS and infarct volume in patients with acute stroke, but there are fewer reports on the degree of disability associated with ischemic volume and NIHSS score [17,18,19,20]. The aim of this study was to determine if the volume of ischemic lesions, as determined with DWI, was an independent predictor of the degree of disability.

Furthermore, this study aimed to demonstrate the reliability and reproducibility of the ABC/2 method for measuring infarct volume using the DWI sequence. Our secondary objective was to correlate the ABC/2 method with the clinical severity of stroke measured by NIHSS and with short- and medium-term prognosis, measured by mRS.

## 2. Materials and Methods

### 2.1. Study Design and Setting

We conducted a monocentric prospective observational study at the largest hospital in Gorj County, which provides secondary care services to a population exceeding 300,000 inhabitants. Ethical approval was obtained from the institutional review board of the hospital (Approval No. 41050/7 November 2022).

### 2.2. Patient Enrollment

Following the application of predefined inclusion and exclusion criteria, we enrolled a total of 43 consecutive patients diagnosed with acute ischemic stroke, whose symptoms had onset within 24 h prior to admission. The enrollment period spanned from January 2023 to December 2023, and patients were admitted to our neurology department for further evaluation and management.

### 2.3. Inclusion Criteria

Diagnosis of acute ischemic stroke: patients diagnosed with acute ischemic stroke confirmed by clinical assessment and imaging.

Onset of symptoms within 24 h: patients whose stroke symptoms had onset within 24 h prior to admission. This criterion was chosen to ensure that we capture the acute phase of stroke, which is critical for evaluating the initial severity and early intervention outcomes.

Age 18 years and older: adult patients were included to ensure the study population is representative of the typical adult stroke population.

Calculation of the NIHSS score: patients in whom it is possible to calculate the NIHSS score.

MRI within 72 h: patients who undergo a brain MRI within the first 72 h of admission showing acute cerebral ischemia in the anterior circulation.

### 2.4. Exclusion Criteria

Posterior territory strokes: Patients with ischemic stroke in the posterior territory were excluded due to insufficient evidence in the literature establishing a clear relationship between ASPECTS score in the posterior territory (ASPECTS-pc) and stroke severity quantified by NIHSS score. Excluding these cases helps maintain the focus on more well-established correlations.

Lacunar infarcts: patients with lacunar infarcts were excluded because the lesion size in lacunar ischemic stroke has been shown to poorly correlate with clinical severity measured by the NIHSS score or short-term prognosis quantified by mRS. This exclusion ensures that our study focuses on ischemic strokes where volume and ASPECTS score correlations are more predictive.

Pre-existing disability: patients with a pre-stroke mRS score greater than 2 were excluded to avoid confounding the assessment of stroke-related disability and prognosis.

Non-contiguous lesions: in cases where two or more non-contiguous lesions were identified, they were classified as involving multiple vascular territories and were excluded. This criterion was chosen to maintain consistency in measuring and correlating stroke volumes and outcomes.

Transient ischemic attack (TIA): patients with TIA, defined as complete disappearance of symptoms within 24 h from onset and no evidence of lesion on MRI, were excluded.

Hemorrhagic conditions: patients whose brain CT or MRI shows hemorrhage (intraparenchymal, subarachnoid, sub/extra-dural), tumor, hemorrhagic contusions, lacerations, or demyelinating lesions were excluded.

Lacunar ischemic lesion visibility: patients with an acute lacunar ischemic lesion were excluded due to the poor correlation between lesion size and clinical severity in such cases (e.g., small lesion, high NIHSS score in pure motor hemiplegia).

Posterior territory ischemic lesions: patients with acute ischemic lesions in the posterior territory were excluded due to the lack of a valid ASPECTS score for this area.

These inclusion and exclusion criteria were carefully selected to ensure a homogeneous study population and to enhance the reliability and validity of our findings. By focusing on acute ischemic strokes with clearly defined criteria, we aimed to minimize variability and better isolate the factors influencing stroke severity and prognosis.

### 2.5. Statistical Analysis

All statistical analyses were performed using SPSS version 25.0, R version 4.0. Continuous variables were expressed as means and standard deviations or medians and interquartile ranges, depending on the distribution of the data. Categorical variables were presented as frequencies and percentages.

To assess the normality of the data, we conducted Kolmogorov–Smirnov tests for the key variables: NIHSS scores, MRI-DWI volume, DWI-ASPECTS scores, and mRS scores. The results indicated that the distribution of these variables did not significantly deviate from normality:

NIHSS scores: D(43) = 0.12, *p* = 0.529

MRI-DWI volume: D(43) = 0.18, *p* = 0.125

DWI-ASPECTS scores: D(43) = 0.19, *p* = 0.0864

mRS scores: D(43) = 0.16, *p* = 0.206

These findings suggest that the sample distributions are not significantly different from a normal distribution, validating the use of parametric statistical tests for subsequent analyses.

### 2.6. Imaging and Clinical Assessment

In all patients, a native brain CT scan was conducted upon admission, and the NIHSS score was computed. Subsequently, within the first 72 h following admission, a comprehensive brain MRI examination was performed, comprising T1-weighted, T2-weighted, FLAIR, DWI + ADC, and susceptibility-weighted imaging (SWI), as well as magnetic resonance angiography (MRA) sequences. Imaging was acquired using a Signa Explorer 1.5 T (GE Healthcare, Tianjin, China) MRI Scanner.

**Figure 1:**
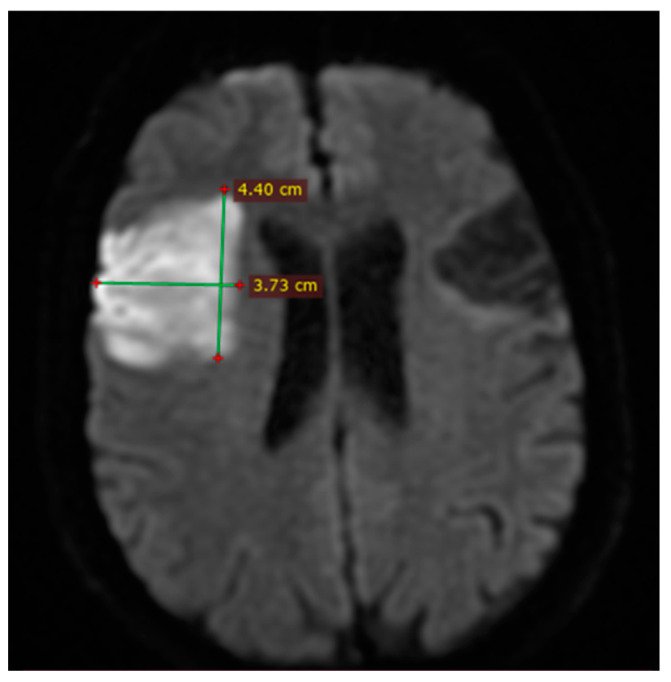
MRI DWI axial section. Example of measurement of stroke volume using ABC/2 formula.

The DWI-ASPECTS score was calculated by a collaborative effort between a radiologist and a neurologist, assessing areas of hyperintensity within each of the 10 ASPECTS regions, as per the established methodology. Stroke volume was determined utilizing the ABC/2 formula, where A represents the largest cross-sectional diameter of the lesion, B represents the perpendicular diameter to A, and C represents the number of slices in which the lesion is visible (Figure 1). This comprehensive imaging approach allowed for precise evaluation of ischemic stroke characteristics and facilitated accurate assessment of stroke volume.

This formula offers a reproducible and highly accurate method for providing a simple geometric estimation of infarct size [21]. Its straightforward application facilitates precise quantification of stroke volume, enhancing clinical assessment and treatment planning.

Moreover, the formula exhibits a high predictive value in identifying the mismatch between DWI and mean transit time (MTT). This mismatch, defined as a MTT/DWI ratio ≥ 1.2, serves as a crucial indicator of perfusion abnormalities and ischemic penumbra, informing therapeutic decisions and prognostic assessments [22]. Such predictive capability underscores the formula’s utility as a valuable tool in elucidating the pathophysiological dynamics of ischemic stroke and guiding optimal patient management strategies.

Patients presenting with acute ischemic stroke in the posterior territory were excluded from the study due to insufficient evidence in the literature establishing a clear relationship between the ASPECTS score value in the posterior territory (ASPECTS-pc) and stroke severity quantified by NIHSS score. Similarly, patients with lacunar lesions were also excluded, as lesion size in lacunar ischemic stroke has been shown to poorly correlate with clinical severity measured by the NIHSS score or short-term prognosis quantified by mRS [23].

In cases where two or more non-contiguous lesions were identified, they were classified as involving multiple vascular territories. For patients with two or more patchy lesions within the same arterial territory, the volume of ischemic tissue was calculated for each lesion separately, and their sum yielded the total volume. This approach ensured a comprehensive assessment of ischemic lesion burden and provided valuable insights into the distribution and severity of stroke pathology.

We investigated the interrelationship among the NIHSS scale, DWI-ASPECTS score, ischemic stroke volume measured with DWI, and mRS score. Spearman’s correlation coefficient was computed to assess the association between the NIHSS score and ischemic stroke volume measured with DWI, as well as between DWI-ASPECTS and mRS score. Additionally, Pearson’s correlation coefficient was calculated to evaluate the correlation between the NIHSS score and the DWI-ASPECTS score, as well as between the NIHSS score and mRS score.

A multiple regression analysis was conducted, with the NIHSS score serving as the independent variable and the ischemic stroke volume measured with DWI and DWI-ASPECTS score as dependent variables. This analysis aimed to elucidate the relationships between stroke severity assessed by the NIHSS score and the extent of ischemic lesion burden as measured by DWI volume and DWI-ASPECTS score.

Furthermore, considering findings from previous large-scale studies where a DWI-ASPECTS score < 4 or ≥7 has been proposed as a reliable proxy for DWI volumes > 100 mL or <70 mL, respectively, we sought to determine if these thresholds also held true within our study cohort [24]. This exploration aimed to validate the utility of DWI-ASPECTS as an indicator of ischemic lesion volume in our specific patient population, providing valuable insights into the clinical relevance of DWI-ASPECTS scoring in assessing stroke severity and prognosis.

### 2.7. Inter-Rater Reliability Analysis

To assess the inter-rater reliability of the ABC/2 formula in calculating infarct volumes, we involved two observers: a radiologist and a neurologist. Both observers independently calculated the infarct volumes using the ABC/2 method. We then analyzed the mean absolute volume differences between the two observers and computed the intraclass correlation coefficient (ICC) to quantify the reliability of the measurements.

### 2.8. Inter-Rater Reliability

The inter-rater reliability analysis demonstrated a mean absolute volume difference of 29.95 ± 2.5 mL between the two observers, indicating a high level of agreement. The intraclass correlation coefficient (ICC) was found to be 0.79 (95% confidence interval), which falls within the range considered to be good reliability. These results suggest that the ABC/2 method provides consistent and reproducible measurements of infarct volume when used by experienced clinicians.

## 3. Results

### 3.1. Demographic and Risk Factor Distribution by Gender (Table 1)

This study included a total of 43 patients, with 19 men (44.19%) and 24 women (55.81%). The median age of the participants was 61.5 years, with men having a higher median age (67 years, IQR 53–74) compared to women (56 years, IQR 48–63).

Smoking history: a higher proportion of men reported a history of smoking (25.58%) compared to women (11.62%). Overall, 37.2% of the participants had a history of smoking.

Alcohol consumption: alcohol consumption was more prevalent among men (13.95%) than women (4.65%), with a total of 18.6% of participants reporting alcohol consumption.

Hypertension: hypertension was the most common risk factor, present in 69.76% of the study population. The prevalence was slightly higher in men (39.53%) compared to women (30.23%).

Atrial fibrillation: atrial fibrillation was found in 32.55% of the participants, with a similar distribution between men (18.6%) and women (13.95%).

Hypercholesterolemia: hypercholesterolemia was more common in women (20.93%) than in men (13.95%), with an overall prevalence of 34.88%.

Hypertriglyceridemia: the prevalence of hypertriglyceridemia was higher in women (16.27%) compared to men (6.97%), affecting 23.25% of the total participants.

Mixed dyslipidemia: mixed dyslipidemia was present in 20.93% of the participants, with a slightly higher prevalence in women (11.62%) than in men (9.3%).

Diabetes mellitus: diabetes mellitus was reported in 37.2% of the participants, with women having a higher prevalence (23.25%) compared to men (13.95%).

These findings highlight the significant burden of cardiovascular risk factors among acute ischemic stroke patients, with notable gender differences in the prevalence of certain risk factors such as smoking, alcohol consumption, and various forms of dyslipidemia. The high prevalence of hypertension and diabetes mellitus underscores the importance of managing these conditions to prevent stroke. These data provide a comprehensive overview of the demographic and risk factor profile of the study population, which is essential for understanding the underlying health conditions that may influence stroke outcomes.

### 3.2. Etiology (Table 2)

The etiology of ischemic stroke in our study population was varied. The most common cause was atrial fibrillation (32.55%), followed by ICA stenosis in the cervical region (>50%) (16.27%), large vessel occlusion of the MCA (11.62%), and ICA occlusion in the cervical region (9.3%).

### 3.3. Affected Vascular Territories (Table 2)

The majority of strokes affected the MCA territory (65.11%), while 27.9% involved the ACA territory. A small portion of strokes (0.69%) affected the border zone of the ICA (MCA/ACA).

### 3.4. Presenting Symptoms (Table 2)

The most frequent presenting symptom was contralateral motor weakness, observed in 86% of patients. Other common symptoms included eye and head deviation (23.27%), contralateral hemianopsia (18.6%), global aphasia (11.62%), Broca’s aphasia (9.3%), Wernicke’s aphasia (6.97%), and hemineglect and disturbed spatial perception (6.97%).

These data provide a detailed overview of the etiological factors, affected vascular territories, and presenting symptoms in our cohort of ischemic stroke patients. This information is crucial for understanding the clinical presentation and underlying causes of strokes in this population, guiding targeted interventions and management strategies.

**Figure 2:**
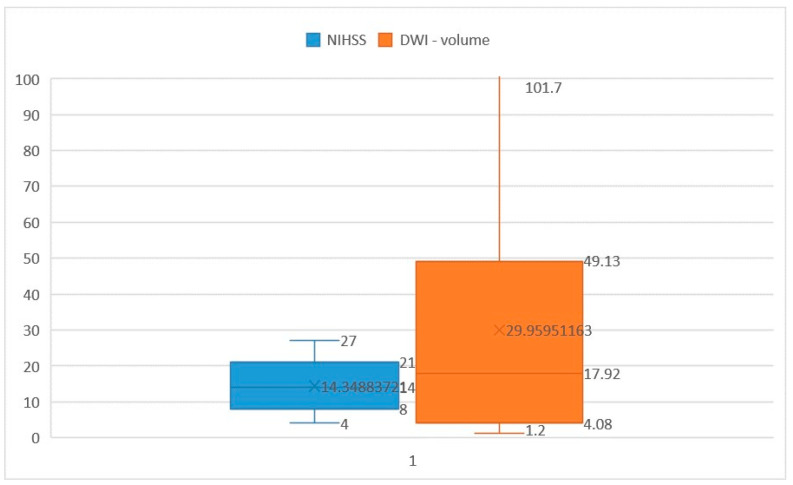
Minimum, maximum and mean values of NIHSS score and ischemic stroke volume calculated with DWI.

Among the patients included in this study, NIHSS scores ranged from 4 to 27 points, with a mean score of 14.34 points (interquartile range [IQR] 21–8 = 13, median [Q2] = 14). Ischemic core volumes varied from 1.2 cm^3^ to 101.7 cm^3^, with a median volume of 29.95 cm^3^ (IQR 49.13–4.08 = 45.05, Q2 = 17.92) (Figure 2). While the close relationship between NIHSS score and ischemic volume assessed by CTP is well-established [25], there exist controversial findings in the literature regarding the correlation between NIHSS score and ischemic stroke volume measured in diffusion imaging [26,27,28,29].

In our study, we observed a Spearman correlation coefficient of 0.982 (*p* < 0.01), indicating a strong positive relationship between these two variables. This finding highlights the robust association between NIHSS score and ischemic stroke volume measured with diffusion imaging within our study cohort, underscoring the utility of NIHSS as a clinical tool for assessing stroke severity and predicting lesion burden.

**Figure 3:**
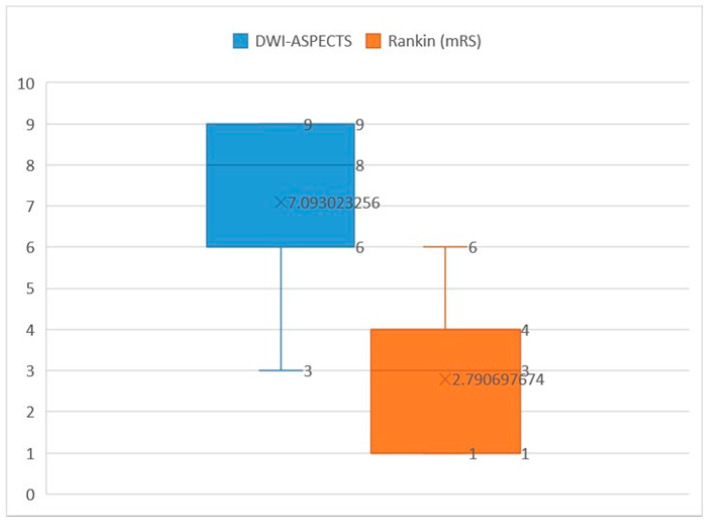
Minimum, maximum, and mean values of DWI-ASPECTS and Rankin.

The mean DWI-ASPECTS score in our study cohort was 7.09 (interquartile range [IQR] 9–6 = 3, median [Q2] = 8). In terms of functional outcomes, the mean Rankin score was 3, with a median of 2.79 (IQR 4–1 = 3, Q2 = 3) (Figure 3). Notably, a strong negative relationship was observed between DWI-ASPECTS and Rankin scores, as evidenced by a Spearman correlation coefficient of −0.952 (*p* < 0.01). This finding underscores the significant association between lower DWI-ASPECTS scores, indicative of greater ischemic lesion burden, and higher Rankin scores, reflecting worse functional outcomes. Thus, DWI-ASPECTS emerges as a valuable tool for predicting functional prognosis in acute ischemic stroke patients, providing clinicians with important insights into the severity of ischemic injury and its impact on patient outcomes.

**Figure 4:**
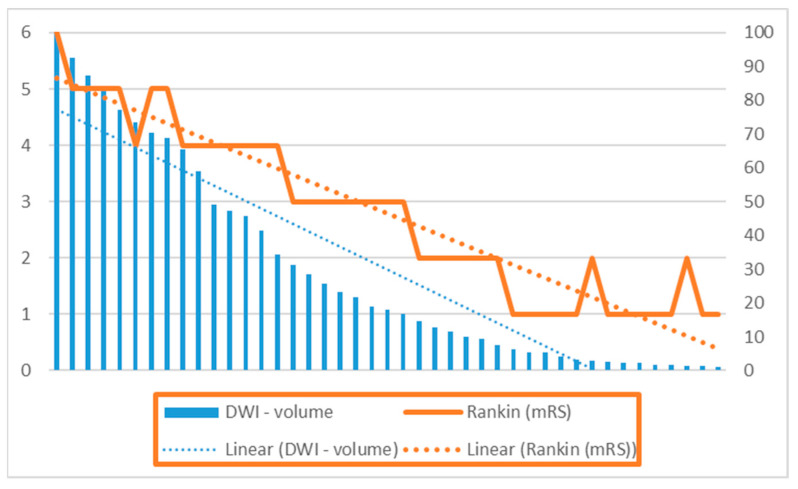
Relationship between DWI and Rankin.

In examining the relationship between ischemic stroke volume measured with DWI and functional outcomes assessed by the Rankin score, our findings indicate that early ischemic changes on diffusion-weighted imaging serve as an independent predictive factor for functional outcome (Figure 4). Notably, previous studies in the literature assessing the relationship between DWI volume of ischemic stroke and prognosis have predominantly utilized the Barthel Disability Scale to measure performance in daily activities, rather than the Rankin score [30].

It is noteworthy that a vast majority of patients in our study cohort (94.5%) had a pre-stroke mRS score of 0, indicating an absence of pre-existing symptoms. Spearman’s correlation coefficient was calculated to explore the relationship between ischemic stroke volume and Rankin score, revealing a significant positive correlation between these two variables (r(41) = 0.953, *p* < 0.001). This robust correlation underscores the prognostic value of ischemic stroke volume measured with DWI in predicting functional outcomes, providing valuable insights into the impact of ischemic lesion burden on post-stroke disability and rehabilitation needs.

In our patient sample, a distinct relationship emerged between DWI-ASPECTS score and DWI-volume. Notably, a volume below 30 mL corresponded with a DWI-ASPECTS score of DWI ≥ 7, while a volume below 70 mL correlated with a DWI-ASPECTS score ≥ 5. Remarkably, only one patient exhibited an ischemic volume exceeding 100 mL, and this individual correspondingly had an ASPECTS score of 3 points.

**Figure 5:**
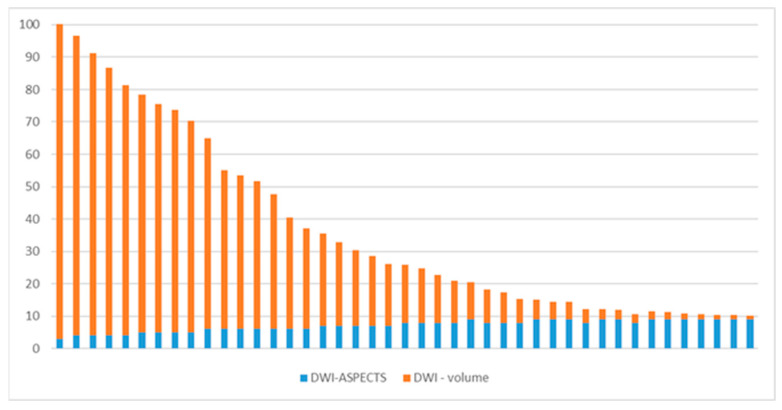
Relationship between DWI-ASPECTS and DWI-volume.

Our analysis revealed a significant negative relationship between ischemic stroke volume in DWI and DWI-ASPECTS score, as depicted in Figure 5 (r(41) = 0.956, *p* < 0.001). This finding underscores the utility of DWI-ASPECTS score as an indicator of ischemic lesion burden, with lower scores reflecting larger ischemic volumes. Such insights are crucial for guiding treatment decisions and prognostic assessments in acute ischemic stroke patients, facilitating more targeted and effective clinical management strategies.

**Figure 6:**
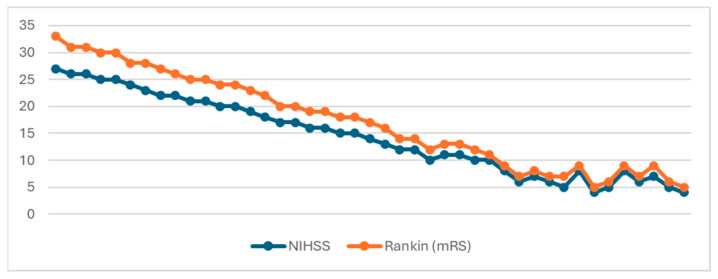
Relationship between NIHSS and mRS.

Upon computing Pearson’s correlation coefficient, our analysis revealed a robust positive relationship between the clinical severity on admission, as indicated by the NIHSS score, and the degree of disability or dependence at discharge quantified by the mRS (r(41) = 0.965, *p* < 0.001) (Figure 6). This finding underscores the predictive value of the NIHSS score in assessing functional outcomes following acute ischemic stroke, with higher NIHSS scores indicative of more severe strokes and poorer functional recovery at discharge. Such insights are pivotal for prognostic assessments and treatment planning, guiding healthcare professionals in optimizing patient care and rehabilitation strategies to enhance post-stroke outcomes.

**Figure 7:**
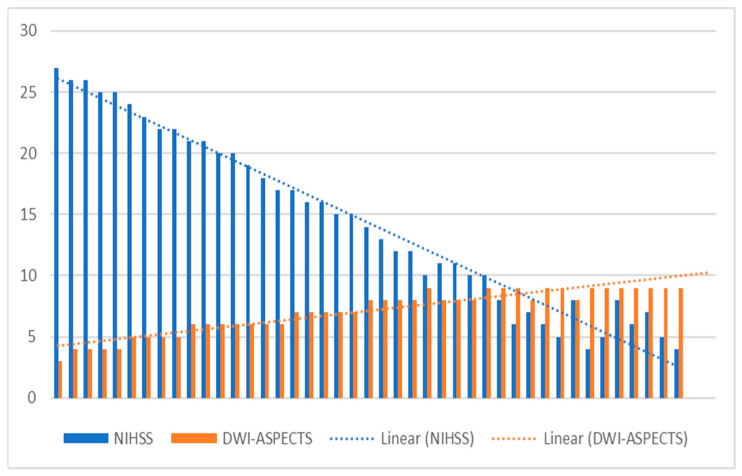
Relationship between NIHSS score and DWI ASPECTS.

Following the calculation of Pearson’s correlation coefficient, our analysis revealed a robust negative relationship between the clinical severity of stroke, quantified by the NIHSS score, and the ASPECTS score calculated with DWI (r(41) = 0.964, *p* < 0.001). This observation underscores a very strong inverse relationship between these two variables (R = −0.9675), as depicted in Figure 7.

Furthermore, the results of multiple linear regression analysis demonstrated a highly significant collective effect between DWI-ASPECTS, DWI-volume and NIHSS score (F(1, 41) = 600.28, *p* < 0.001, R^2^ = 0.94, R^2^_adj_ = 0.93). This finding underscores the comprehensive influence of these variables on stroke severity and lesion burden, providing valuable insights into the multifaceted nature of ischemic stroke pathology and its clinical manifestations. Such insights are essential for guiding treatment decisions and prognostic assessments, facilitating tailored interventions to optimize patient outcomes in acute ischemic stroke management.

### 3.5. Translation of Research Findings into Clinical Practice

Our study highlights several key correlations between clinical severity, ischemic stroke volume, and functional outcomes that have important implications for clinical practice.

NIHSS Score and Ischemic Volume: The strong positive correlation between NIHSS score and ischemic stroke volume measured with DWI underscores the utility of the NIHSS as a reliable tool for assessing stroke severity. This correlation can guide clinicians in quickly identifying patients with large ischemic volumes who may benefit from more aggressive interventions, such as mechanical thrombectomy, especially beyond the traditional therapeutic window as indicated by studies like the DAWN trial.

DWI-ASPECTS Score and Functional Outcomes: The significant negative correlation between DWI-ASPECTS scores and Rankin scores suggests that DWI-ASPECTS can be a valuable predictor of functional prognosis. Lower DWI-ASPECTS scores, indicating larger ischemic lesion burdens, are associated with worse functional outcomes. Incorporating DWI-ASPECTS into routine clinical assessment can help clinicians predict patient outcomes more accurately and tailor rehabilitation strategies accordingly.

Ischemic Stroke Volume and Disability: The robust correlation between ischemic stroke volume in DWI and post-stroke disability, as measured by the mRS, emphasizes the prognostic value of early imaging in predicting long-term functional outcomes. By identifying patients at higher risk for severe disability, healthcare providers can initiate early and intensive rehabilitation programs, potentially improving recovery and reducing long-term care needs.

Integrated Use of Imaging and Clinical Scores: Our findings support the integrated use of clinical scores (NIHSS, mRS) and imaging metrics (DWI volume, DWI-ASPECTS) to provide a comprehensive assessment of stroke severity and prognosis. This approach can enhance decision-making regarding the most appropriate and timely interventions, optimizing patient management.

Guiding Treatment Decisions: The clear relationships between imaging scores and clinical outcomes can guide treatment decisions. For instance, patients with high NIHSS scores and large ischemic volumes may be prioritized for thrombectomy and other advanced treatments. Similarly, understanding the likely functional outcomes can help in setting realistic goals and expectations for patients and their families, improving overall care satisfaction.

## 4. Discussion

While not yet standard in diagnostic protocols, the DWI-ASPECTS score and DWI volume of ischemic stroke are progressively gaining traction among radiologists and neurologists for descriptive and prognostic purposes. Assessing stroke volume with DWI sequences holds particular significance, aiding clinicians in gauging the extent of stroke involvement and guiding acute-phase treatment decisions such as intravenous thrombolysis or mechanical thrombectomy. Consequently, DWI volume serves as a valuable predictive factor for stroke prognosis, offering clinicians crucial insights into patient outcomes and informing tailored therapeutic strategies for acute ischemic stroke management.

The clinical significance of the DWI sequence has escalated with the advent of revascularization procedures. Notably, a large pre-treatment lesion volume observed using DWI is strongly linked to a poor prognosis, often indicating a more severe stroke and correlating with a heightened degree of neurological deficit [31]. Conversely, smaller volumes detected with DWI typically herald a more favorable functional outcome, often leading to earlier, and sometimes complete, neurological recovery, particularly when timely and appropriate treatment is administered.

Moreover, it is important to recognize that the risk of hemorrhagic transformation of ischemic stroke escalates with increasing volume in the DWI sequence [32,33]. This underscores the critical role of DWI imaging in risk stratification and treatment decision-making, emphasizing the imperative of promptly identifying and managing stroke patients with varying degrees of lesion burden to optimize outcomes and minimize complications.

### Limitations

Certainly, our study has several limitations that need to be addressed. Firstly, being conducted at a single institution with a relatively small cohort of patients, the study lacks the statistical power to yield definitive conclusions. This limitation affects the generalizability of our findings. Despite this, certain trends were observed, suggesting that increasing the sample size in future studies could help identify independent predictors of prognosis using more robust regression models.

Secondly, the DWI-ASPECTS score and ischemic stroke volume were manually calculated by a radiologist in conjunction with a neurologist. This manual process is time-consuming and susceptible to potential errors and variability depending on the examiner’s experience. Future research should consider incorporating automated or semi-automated methods for these calculations to improve efficiency and standardize assessments across different examiners, reducing potential bias and variability.

Additionally, formal sample size calculation and power analysis were not conducted prior to this study. Our research was designed as a monocentric prospective observational study, primarily intended to explore and describe the relationships among NIHSS scores, ischemic stroke volume, DWI-ASPECTS scores, and short-term prognosis within a defined patient cohort. We acknowledge that the lack of a priori sample size and power calculations is a limitation of our study. To address this, we recommend that future research in this area incorporate these elements to ensure robust and generalizable findings. Despite this limitation, our study provides valuable preliminary insights and establishes a foundation for more extensive research.

Lastly, while our study provides valuable insights into the correlation between clinical severity, ischemic stroke volume, and short-term prognosis, it is essential to acknowledge that these findings need to be validated in larger, multicenter studies. Such studies would help to confirm our results and potentially uncover additional factors that influence the prognosis of ischemic stroke patients.

While our study has contributed to the understanding of the relationships among DWI-ASPECTS score, ischemic stroke volume, and clinical outcomes, addressing these limitations in future research will be critical for enhancing the accuracy and applicability of these findings in clinical practice.

## 5. Conclusions

In conclusion, our study has illuminated several critical aspects regarding the utility of measuring ischemic stroke volume and ASPECTS score in the DWI sequence:

Relationship with Clinical Severity: We have established a strong positive relationship between the clinical severity of ischemic stroke, as quantified by the NIHSS score, and the ischemic stroke volume measured with DWI.

Influence on Short-Term Prognosis: The short-term prognosis of patients, as quantified by the mRS score, is significantly influenced by the ischemic stroke volume measured in the DWI sequence.

Utility of ASPECTS Score: The ASPECTS score measured in the DWI sequence emerges as a valuable and reliable tool for assessing both the clinical severity of ischemic stroke and prognosis.

Volume Correlations: Notably, an ischemic stroke volume below 30 mL correlates with an ASPECTS score of DWI ≥ 7, while a volume below 70 mL correlates with an ASPECTS score ≥ 5. Volumes below 30 mL are associated with a Rankin score ≤ 3, indicating a moderate outcome where patients still retain some degree of mobility.

These findings underscore the clinical relevance and prognostic significance of DWI-based assessments in acute ischemic stroke, providing clinicians with valuable insights for guiding treatment decisions and prognostic assessments.

### Future Research Directions

Longitudinal Studies on Stroke Outcomes: Future research should focus on longitudinal studies to track the long-term outcomes of patients with varying ischemic volumes and NIHSS scores. This would provide deeper insights into the chronic impact of ischemic stroke and the effectiveness of different rehabilitation strategies over time.

Advanced Imaging Techniques: Investigating the use of more advanced imaging techniques, such as perfusion MRI and CT perfusion, in conjunction with DWI and ASPECTS scores, could enhance the precision of ischemic core and penumbra estimation. This could lead to more targeted treatment approaches and better prediction of treatment outcomes.

Biomarkers and Genetic Studies: Exploring biomarkers and genetic factors that correlate with ischemic stroke severity and recovery could offer new avenues for personalized medicine. Identifying patients who are genetically predisposed to worse outcomes could lead to tailored therapeutic interventions and preventative strategies.

Interventional Studies: Conducting randomized controlled trials to compare the efficacy of different treatment modalities, such as thrombolysis, thrombectomy, and neuroprotective agents, in patients stratified by NIHSS and DWI-ASPECTS scores. This could refine treatment guidelines and improve patient-specific treatment plans.

Functional Outcome Predictors: Investigating additional predictors of functional outcomes, such as cognitive and psychological assessments, in conjunction with traditional scales like mRS. Understanding the full spectrum of post-stroke disability could enhance holistic patient care.

Telemedicine and Remote Monitoring: Exploring the role of telemedicine and remote monitoring technologies in the follow-up and management of stroke patients. Studies should assess the impact of these technologies on patient outcomes, particularly in underserved or remote areas.

Future research in these areas could significantly enhance our understanding of ischemic stroke and its management, leading to better patient outcomes and more efficient healthcare delivery. By building on the correlations identified in this study, researchers can develop more precise and personalized approaches to stroke care.

## Figures and Tables

**Table 1:** Demographic and risk factor distribution by gender.

Characteristic	Men (n = 19)	Women (n = 24)	Total (n = 43)
Median age in years	67 (IQR 53–74)	56 (IQR 48–63)	61.5 (IQR 50–69)
Smoking history	11 (25.58%)	5 (11.62%)	16 (37.2%)
Alcohol consumption	6 (13.95%)	2 (4.65%)	8 (18.6%)
Hypertension	17 (39.53%)	13 (30.23%)	30 (69.76%)
Atrial fibrillation	8 (18.6%)	6 (13.95%)	14 (32.55%)
Hypercholesterolemia	6 (13.95%)	9 (20.93%)	15 (34.88%)
Hypertrigliceridemia	3 (6.97%)	7 (16.27%)	10 (23/25%)
Mixed dyslipidemia	4 (9.3%)	5 (11.62%)	9 (20.93%)
Diabetes mellitus	6 (13.95%)	10 (23.25%)	16 (37.2%)

**Table 2:** Etiology, affected vascular territories, and presenting symptoms in the study population.

**Etiology**	
ICA stenosis in cervical region (>50%)	7 (16.27%)
ICA occlusion in cervical region	4 (9.3%)
Large vessel occlusion of MCA	5 (11.62%)
Atrial fibrillation	14 (32.55%)
**Affected territories**	
MCA	28 (65.11%)
ACA	12 (27.9%)
Border zone of ICA (MCA/ACA)	3 (0.69%)
**Presenting symptoms**	
Contralateral motor weakness	37 (86%)
Contralateral hemianopsia	8 (18.6%)
Global aphasia	5 (11.62%)
Broca’s aphasia	4 (9.3%)
Wernike’s aphasia	3 (6.97%)
Hemineglect and disturbed spatial perception	3 (6.97%)
Eye and head deviation	11 (23.27%)

## Data Availability

The original contributions presented in the study are included in the article, further inquiries can be directed to the corresponding author.
